# Correlation between cardiopulmonary metabolic energy cost and lower-limb muscle activity during inclined treadmill gait in older adults

**DOI:** 10.1186/s12877-021-02401-9

**Published:** 2021-08-23

**Authors:** Jihye Kim, Hwang-Jae Lee, Su-Hyun Lee, Jungsoo Lee, Won Hyuk Chang, Gyu-Ha Ryu, Yun-Hee Kim

**Affiliations:** 1 Department of Physical and Rehabilitation Medicine, Center for Prevention and Rehabilitation, Heart Vascular Stroke Institute, Samsung Medical Center, Sungkyunkwan University School of Medicine, 81 Irwon-ro, Gangnam-gu, Seoul, 06351 Republic of Korea; 2 Department of Health Science and Technology, Department of Digital Health, SAIHST, Sungkyunkwan University, 81 Irwon-ro, Gangnam-gu, Seoul, 06351 Republic of Korea; 3 Department of Medical Device Management and Research, SAIHST, Sungkyunkwan University, 81 Irwon-ro, Gangnam-gu, Seoul, 06351 Republic of Korea

**Keywords:** Aged, Walking, Oxygen consumption, Electromyography, Lower extremity

## Abstract

**Background:**

Inclined walking requires more cardiopulmonary metabolic energy and muscle strength than flat-level walking. This study sought to investigate changes in lower-limb muscle activity and cardiopulmonary metabolic energy cost during treadmill walking with different inclination grades and to discern any correlation between these two measures in older adults.

**Methods:**

Twenty-four healthy older adults (*n* = 11 males; mean age: 75.3 ± 4.0 years) participated. All participants walked on a treadmill that was randomly inclined at 0% (condition 1), 10% (condition 2), and 16% (condition 3) for five minutes each. Simultaneous measurements of lower-limb muscle activity and cardiopulmonary metabolic energy cost during inclined treadmill walking were collected. Measured muscles included the rectus abdominis (RA), erector spinae (ES), rectus femoris (RF), biceps femoris (BF), vastus medialis (VM), tibialis anterior (TA), medial head of the gastrocnemius (GCM), and soleus (SOL) muscles on the right side.

**Results:**

As compared with 0% inclined treadmill gait, the 10% inclined treadmill gait increased the net cardiopulmonary metabolic energy cost by 22.9%, while the 16% inclined treadmill gait increased the net cardiopulmonary metabolic energy cost by 44.2%. In the stance phase, as the slope increased, activity was significantly increased in the RA, RF, VM, BF, GCM, and SOL muscles. In the swing phase, As the slope increased activity was significantly increased in the RA, RF, VM, BF, and TA muscles. SOL muscle activity was most relevant to the change in cardiopulmonary metabolic energy cost in the stance phase of inclined treadmill walking. The relationship between the increase in cardiopulmonary metabolic energy cost and changes in muscle activity was also significant in the VM, GCM, and RF.

**Conclusion:**

This study demonstrated that changes in SOL, VM, GCM, and RA muscle activity had a significant relationship with cardiopulmonary metabolic energy cost increment during inclined treadmill walking. These results can be used as basic data for various gait-training programs and as an indicator in the development of assistive algorithms of wearable walking robots for older adults.

**Trial registration:**

Clinical trials registration information: ClinicalTrials.gov Identifier: NCT04614857 (05/11/2020).

## Background

The proportion of the population aged 65 years or older has increased dramatically in recent decades, increasing from 6% globally in 1990 to 9% in 2019. This proportion is projected to rise further to 16% by 2050 [1]. Therefore, the increase in the proportion of older adults is emerging as an important social issue worldwide, which has led to greater interest in the health characteristics of these individuals.

Aging involves the gradual degeneration of the structure and function of the physical body with time. Physical changes due to aging, exposure to chronic diseases, lifestyle changes, and reduced activity deteriorate the body and gait functions [2–4]. Normal individuals move their bodies forward by alternating stance and swing phases with a rhythmic gait cycle and periodic, repetitive gait movement. To support normal gait, good postural control, weight-bearing, efficient rhythmic movement pattern, and selective timing of muscle activity during repeated gait cycles are required [5]. Among older adults, gait function is important for daily life tasks, and changes in gait function can predict clinical abnormalities [6]. The typical gait of an older person is characterized by a slower speed, short stride, and reduced range of motion relative to young adults; however, the relative activation of lower limb muscles during walking is greater in older people [7]. Furthermore, older adults require greater muscle activity compared to young adults to maintain a fast walking speed [8]. According to a previous study, lower-limb muscle activity increases during walking with advancing age, and there is a correlation between increased lower-limb muscle activity and an inability to balance the body [9].

In older adults, walking ability gradually decreases and energy consumption increases when encountering environmental obstacles such as slopes during activities of daily living. Older adults are at greater risk of injury than younger adults due to kinematic differences between older and younger adults when walking uphill [10]. Inclined walking requires more muscle strength and energy than flat-level walking. Older adults show much more muscle activity when walking on an incline relative to younger adults [11]. While walking on a flat surface or uphill for a given distance, older adults spend 7 to 20% more metabolic energy than younger adults [7]. In a previous study of young adults, the pattern of muscle activity during inclined walking was different from that of flat-level walking, and increased muscle activity was associated with a rise in cardiopulmonary metabolic energy consumption [12]. However, the relationship between muscle activity pattern and cardiopulmonary metabolic energy cost under inclined gait conditions in older adults has not been studied sufficiently.

This study aimed to confirm the correlation between the activity of lower limb muscles and the cardiopulmonary metabolic energy cost in older adults during inclined treadmill walking by measuring both the cardiopulmonary metabolic energy cost and lower-limb muscle activity simultaneously. In addition, we aimed to identify the activity of individual muscles that show a strong correlation with cardiopulmonary metabolic energy cost during inclined walking as the specific muscles involved would be potential targets for intervention to improve the gait efficiency of older adults. There are three main hypotheses as follows: (1) inclined treadmill gait induces changes in the cardiopulmonary metabolic energy cost in older adults according to the slope, (2) lower-limb muscle activity change during inclined treadmill gait in older adults according to the slope, and (3) there is a correlation between cardiopulmonary metabolic energy cost and lower-limb muscle activity during inclined treadmill gait in older adults.

## Methods

### Participants

Twenty-four healthy older adults who met the following inclusion criteria were enrolled in this study: (1) age of 65 to 84 years with no history of central nervous system disease, (2) the ability to walk on a 16% slope, and (3) moderate performance score of eight points or higher on the Short Physical Performance Battery (SPPB) test [13]. Study exclusion criteria were (1) uncontrolled severe high blood pressure or diabetes, (2) history of uncontrolled cardiovascular disease, (3) acute injury such as fracture or prior orthopedic surgery such as artificial joint replacement within the 6 months preceding the study, (4) severe dizziness that might lead to a fall, and (5) cognitive disorders that hinder the ability to understand or comply with study instructions. Study participants’ general characteristics are summarized in Table 1. All subjects provided informed consent before participating in this study and the study protocol was approved by the institutional review board of Samsung Medical Center, Seoul, Korea (no. 2020-08-147).

**Table 1:** Characteristics of the study participants

Characteristics	Value
Gender (male/female)	11/13
Age (*years*)	75.33 (4.03)
Height (*cm*)	160.38 (7.04)
Weight (*kg*)	61.38 (8.79)
Body mass index (*kg/m*^*2*^)	23.77 (2.11)
Short Physical Performance Battery (total score: 12 points)	11.33 (0.91)
Treadmill speed (*km/h*)	2.22 (0.19)

Continuous values are presented as mean (standard deviation)

### Experimental protocol

**Fig. 1:**
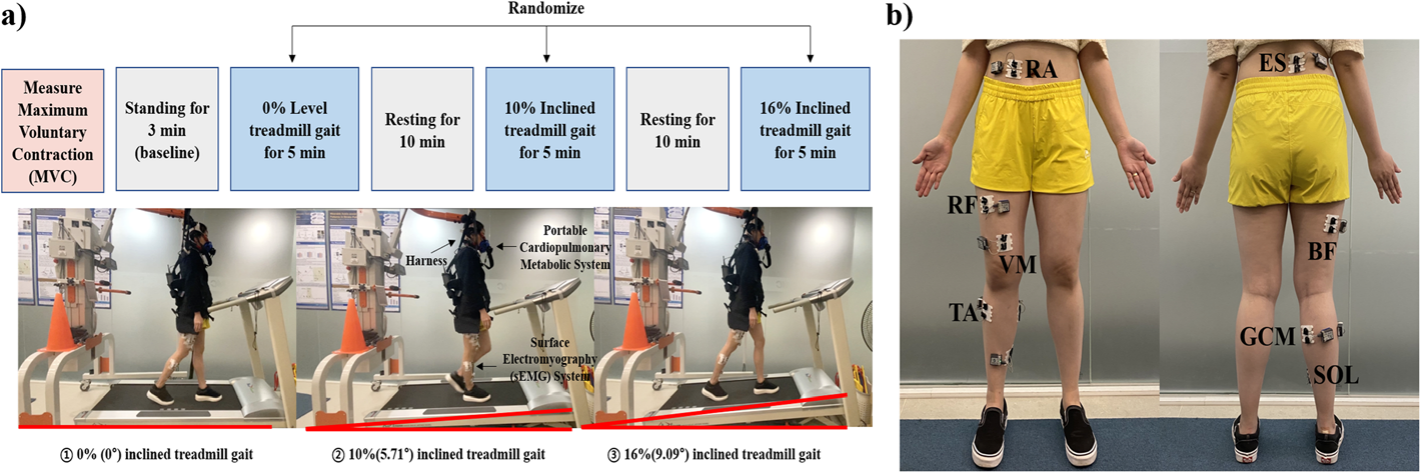
**a)** Experimental protocol. **b)** Measured muscles. RA, rectus abdominis; ES, erector spinae; RF, rectus femoris; BF, biceps femoris; VM, vastus medialis; TA, tibialis anterior; GCM, medial head of the gastrocnemius; SOL, soleus

Prior to the experimental trial, basic physical details (age, gender, height, body weight, and blood pressure), medical history, and pain level were recorded; also, the physical performance abilities of study participants were measured with the SPPB to ensure compliance with the inclusion criteria. Then, the cardiopulmonary metabolic energy cost and muscle activation of all participants were evaluated on an inclined treadmill at 0% (condition 1), 10% (condition 2), and 16% (condition 3) for five minutes each in a random order. Randomization sequences were generated using an online website (www.randomization.com) to determine sequences of the inclined treadmill gait conditions. Participants’ self-selected gait speed was determined after walking for three minutes on a 0% inclined treadmill. Adequate rest for 10 min was given between each walking condition; an additional five minutes was allowed if the participant requested more rest time (Fig. 1). A harness that did not provide weight support was used as a safety device to prevent falls. In addition, safety personnel and physical therapists supervised the study to ensure immediate availability in the event of an emergency. For the simultaneous measurement of muscle activity and cardiopulmonary metabolic energy cost during inclined treadmill gait, a multichannel surface electromyography (sEMG) system and a portable metabolic analyzer were used. Metabolic energy expenditure was measured using a portable cardiopulmonary metabolic system (K5; COSMED, Rome, Italy) based on actual breathing during each inclined treadmill gait condition. Muscle activity was measured using an sEMG system (TeleMyo Desktop DTS; Noraxon, Scottsdale, AZ, USA) during each inclined treadmill gait condition.

### Measurement equipment

#### Portable cardiopulmonary metabolic system

The COSMED K5 wearable metabolic system was used to measure metabolic energy expenditure during each inclined treadmill gait condition. The COSMED K5 wearable metabolic system works using combined breath-by-breath technology to measure oxygen consumption (VO_2_) and carbon dioxide production (VCO_2_) for physical performance and clinical diagnosis. This device analyzes the amount, flow, and proportions of oxygen and carbon dioxide in the exhaled gas. As expired gas is discharged through the turbine, the device senses the volume of respiration that is transmitted to the inside of the device through a sampling line connected to the turbine; information is analyzed by sensors inside the device. To ensure proper operation of the COSMED K5 analyzer device, the flow turbine and gas analyzer were calibrated using a 3-l calibration syringe, gas, and regulators prior to each experiment.

#### sEMG system

The sEMG system (TeleMyo Desktop DTS) was used to measure muscle activity during each inclined treadmill gait condition. The sEMG system is a noninvasive option for measuring physiological muscle signals that are detected by an electrode attached to the skin surface over the muscle of interest. These electrodes measure the bioelectrical signals generated by muscle movement. The signal is measured by synthesizing the motor-unit action potentials generated in the muscles around the surface electrodes. A potential difference is formed between the surface electrode and the spatial distance of the motor units, which is passed through the amplifier and the surface EMG signal is measured and recorded.

### Data collection and analysis

#### Cardiopulmonary metabolic energy cost

Prior to starting inclined treadmill gait measurements, the COSMED K5 portable cardiopulmonary metabolic system was attached to the participant’s upper body. The participant wore a face mask for breath analysis that prevented exposure to outside air. The net metabolic cost during standing and during each inclined gait was calculated using Brockway’s eq. [14]. Baseline values were obtained for all participants by measuring cardiopulmonary metabolic energy cost during a comfortable standing position for three minutes. Then, the cardiopulmonary metabolic energy cost while in gait for five minutes in each of the three incline conditions was measured. The net cardiopulmonary metabolic cost (mL·kg^− 1^·min^− 1^) was calculated by subtracting the last-minute gait data from the baseline. The net energy expenditure measurement (EEm) (Kcal/min) was calculated in the same way.

#### Lower-limb muscle activity

To evaluate lower-limb muscle activation, the percentage of maximum voluntary contraction (MVC) of different muscles during inclined walking was measured prior to starting the inclined treadmill gait. The MVC is the most common method to normalize EMG signals. All participants performed the MVC measurement to obtain the peak value of EMG signals in the trunk and lower-limb muscles before gait assessment. Participants were instructed to push against the examiner as hard as they could, and examiner counteracted that force with both hands for five seconds. To normalize the sEMG signal amplitude, measurements of MVC were collected over five seconds for each muscle to determine the MVC value per muscle for each participant. Electrodes were placed on the right side of the rectus abdominis (RA), the erector spinae (ES), rectus femoris (RF), biceps femoris (BF), vastus medialis (VM), tibialis anterior (TA), medial head of the gastrocnemius (GCM), and soleus muscles (SOL) according to the recommendations of the Surface Electromyography for the Noninvasive Assessment of Muscles Project [15]. In addition, two switch sensors were placed on the right plantar toe and heel surface to record the stance and swing phases during the gait cycle. The stance phase of gait begins when the foot first touches the ground and ends when the same foot leaves the ground. The stance phase makes up approximately 60% of the gait cycle. The swing phase begins when the foot first leaves the ground and ends when the same foot touches the ground again. The swing phase makes up the other 40% of the gait cycle [16]. Muscle activity was measured using the Myo Research XP Master Edition software (Noraxon). The sEMG signal sampling rate was set to 1000 Hz with frequency filtering of 10 to 350 Hz by a band-pass filter. For analysis, a sliding 100-ms window was used to calculate the root mean square value of the signal by gait condition. Inclined walking was normalized to the MVC data obtained during level walking [17].

### Statistical analysis

All data were analyzed using the SPSS version 22.0 program (SPSS Inc., Chicago, IL, USA). Results were calculated as mean and standard deviation values. A one-way analysis of variance was used to compare cardiopulmonary metabolic energy cost and muscle activation according to incline gradient among participants, and the Bonferroni post-test was performed. After normality testing for all variables, the correlation between cardiopulmonary metabolic energy cost and each muscle’s activity in each incline condition was analyzed using Pearson’s correlation analysis. Standardized β-coefficients in the linear regression analysis were used to investigate the relationship between changes in the percentage of MVC of lower limb muscles and the increase in the cardiopulmonary metabolic energy cost according to the degree of treadmill inclination. The significance level was set to *p* < 0.05.

## Results

### Cardiopulmonary metabolic energy cost

**Fig. 2:**
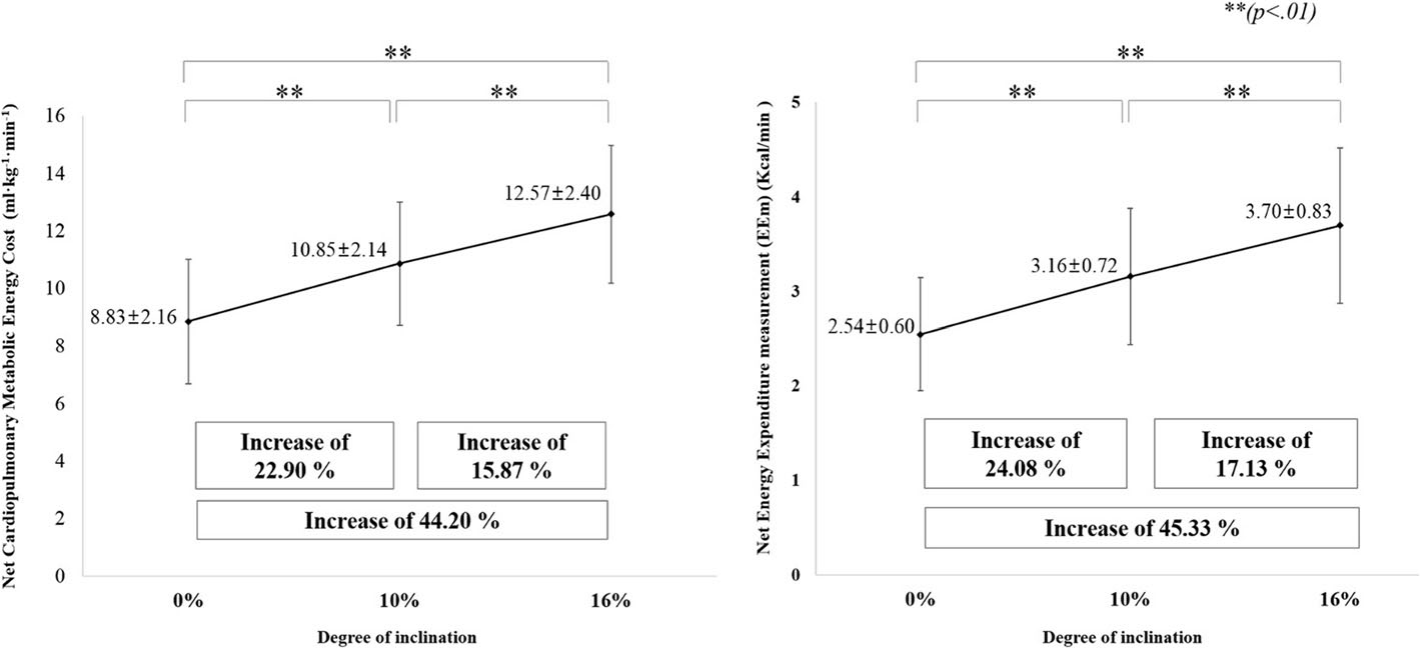
Comparison of average net cardiopulmonary metabolic energy cost and net energy expenditure measurement during 0, 10, and 16% inclined conditions

The obtained measurements confirmed a significant difference among net cardiopulmonary metabolic energy costs (mL·kg^− 1^·min^− 1^) under the three inclined gait conditions (Fig. 2). As compared with a 0% inclined treadmill gait (8.83 ± 2.16 mL·kg^− 1^·min^− 1^), the 10% inclined treadmill gait (10.85 ± 2.14 mL·kg^− 1^·min^− 1^) increased the net cardiopulmonary metabolic energy cost by 22.90% (*p* < 0.01), while the 16% inclined treadmill gait (12.57 ± 2.40 mL·kg^− 1^·min^− 1^) increased the net cardiopulmonary metabolic energy cost by 44.20% (*p* < 0.01). As compared with the 10% inclined treadmill gait, the 16% inclined treadmill gait increased the net cardiopulmonary metabolic energy cost by 15.87% (*p* < 0.01) (Fig. 2). As the slope increased, the net cardiopulmonary metabolic energy cost also rose, and all increases were statistically significant. EEm (Kcal/min) values also showed significant differences among three inclined gait conditions (Fig. 2): relative to the 0% inclined treadmill gait (2.54 ± 0.60 Kcal/min), the 10% inclined treadmill gait was 24.08% greater (*p* < 0.01) (3.16 ± 0.72 Kcal/min) and the 16% treadmill gait was 45.33% greater (*p* < 0.01) (3.70 ± 0.83 Kcal/min). In addition, as compared with the 10% inclined treadmill gait, the 16% inclined treadmill gait increased the net EEm by 17.13% (*p* < 0.01). As the slope increased, both the net cardiopulmonary metabolic energy cost and net EEm increased according to a linear regression model (net cardiopulmonary metabolic energy cost ~ inclination + subject ID, *p* < 0.001; Net EEm ~ inclination + subject ID, *p* < 0.001).

### Lower-limb muscle activity

**Fig. 3:**
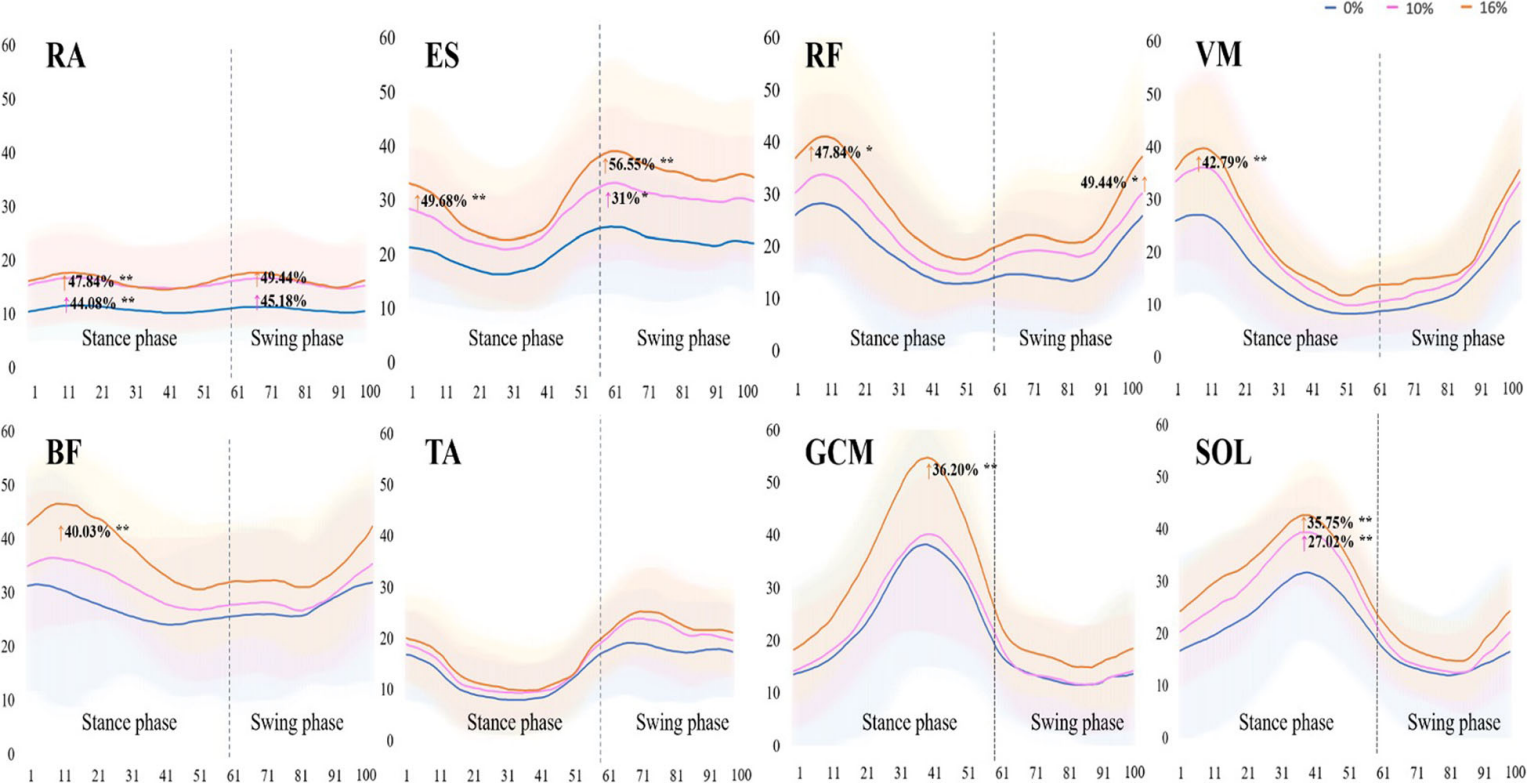
Changes in the percentage of MVC during the gait cycle according to treadmill inclination; RA, rectus abdominis; ES, erector spinae; RF, rectus femoris; BF, biceps femoris; VM, vastus medialis; TA, tibialis anterior; GCM, medial head of the gastrocnemius; SOL, soleus (*p < 0.05, * *p < 0.01)

In all measured muscles, as the inclination increased, muscle activity also tended to increase. However, the statistically significant increase in muscle activation with increasing slope was different for each muscle (Fig. 3). In the stance phase, relative to the 0% inclined treadmill gait (10.89 ± 3.97%MVC), RA muscle activity increased by 44.08% during the 10% inclined treadmill gait (15.69 ± 6.08%MVC) and by 47.84% during the 16% inclined treadmill gait (16.10 ± 6.80%MVC) (*p* < 0.01). In the swing phase, as compared with the 0% inclined treadmill gait (10.78 ± 3.95%MVC), RA muscle activity increased by 45.18% during the 10% inclined treadmill gait (15.65 ± 6.48%MVC) and by 49.44% during the 16% inclined treadmill gait (16.11 ± 6.73%MVC) (*p* < 0.01). The ES muscle activity was significantly increased by 49.68% during the 16% inclined treadmill gait (30.40 ± 9.13%MVC) in the stance phase of the gait cycle (*p* < 0.01). In the swing phase of the gait cycle, as compared with during the 0% inclined treadmill gait (22.81 ± 8.85%MVC), ES muscle activity was increased by 31% during 10% inclined treadmill gait (29.88 ± 11.28%MVC) (*p* < 0.05) and by 56.55% during 16% inclined treadmill gait (35.71 ± 11.98%MVC) (*p* < 0.01). RF muscle activity was significantly increased during 16% inclined treadmill gait in the stance and swing phases of the gait cycle (*p* < 0.05). In the stance phase of the gait cycle, muscle activity for VM, BF, and GCM did not increase significantly in the 10% inclined treadmill gait relative to 0%. However, muscle activity in 16% inclined walking increased significantly (*p* < 0.01). In the SOL stance phase, muscle activity increased statistically significantly during the 10 and 16% inclined treadmill gait as compared with during the 0% inclined treadmill gait (25.65 ± 6.45%MVC). SOL muscle activity was increased by 44.08% during the 10% inclined treadmill gait (32.58 ± 8.74%MVC) and by 47.84% during the 16% inclined treadmill gait (34.82 ± 10.22%MVC) (*p* < 0.01).

According to a linear regression model (%MVC ~ inclination + subject ID) (Table 2), in the stance phase, as the slope increased, the increase in muscle activity was significant in the RA, ES, RF, VM, BF, GCM, and SOL. In the swing phase, the increase in muscle activity was significant in the RA, ES, RF, VM, BF, and TA. Also, the degree of change in each muscle’s activity with increasing inclination was investigated using standardized β-coefficients of a linear regression model. The ES activity showed the greatest increase in the stance and swing phases as the slope increased.

**Table 2:** Changes in percentage of MVC of lower limb muscles according to increasing treadmill inclination

Muscle	Stance phase					Swing phase				
	ß	Std. Error	t	*p*	R^2^	ß	Std. Error	t	*p*	R^2^
RA	0.370	0.788	3.306	0.002**	0.137	0.371	0.803	3.317	0.001**	0.138
ES	0.475	1.132	4.445	0.000***	0.234	0.474	1.453	4.434	0.000***	0.234
RF	0.311	1.445	2.718	0.008**	0.097	0.324	1.313	2.843	0.006**	0.106
VM	0.297	1.281	2.593	0.012*	0.096	0.252	1.085	2.182	0.033*	0.082
BF	0.446	1.272	4.161	0.000***	0.220	0.244	1.295	2.200	0.031*	0.148
TA	0.189	0.697	1.700	0.094	0.163	0.257	1.276	2.193	0.032*	0.066
GCM	0.326	1.318	2.849	0.006**	0.147	0.161	1.244	1.364	0.177	0.035
SOL	0.427	1.137	4.033	0.000***	0.227	0.231	1.245	1.971	0.053	0.054

*RA* rectus abdominis, *ES* erector spinae, *RF* rectus femoris, *BF* biceps femoris, *VM* vastus medialis, *TA* tibialis anterior, *GCM* medial head of the gastrocnemius, *SOL* soleus

*p < 0.05, **p < 0.01, ***p < 0.001

### Relationship between cardiopulmonary metabolic energy cost and lower-limb muscle activity

**Fig. 4:**
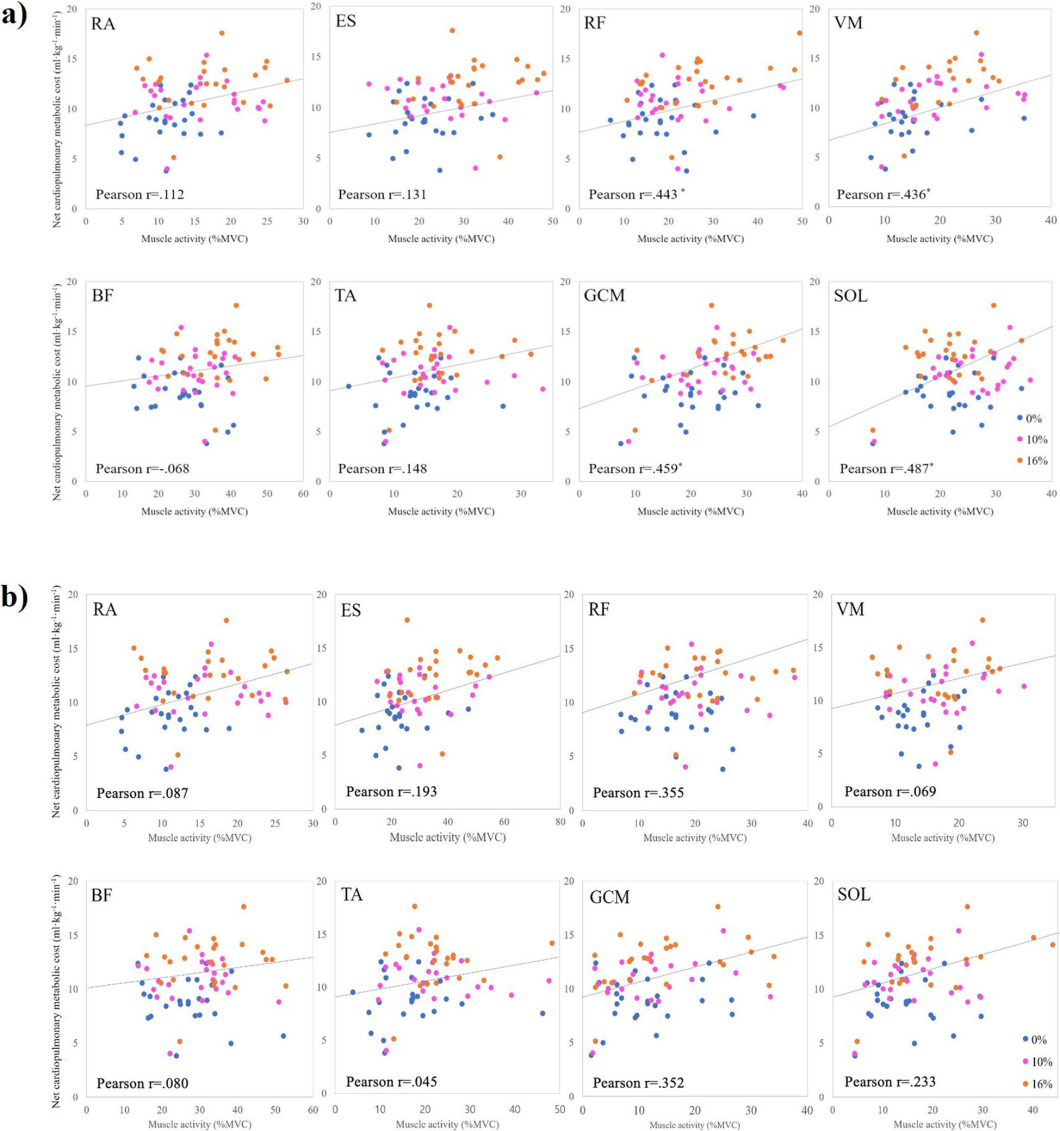
**a)** Correlation between the net cardiopulmonary metabolic energy cost and percentage of MVC in the stance phase of the gait cycle during inclined treadmill walking (*p < 0.05). **b)** Correlation between net cardiopulmonary metabolic energy cost and the percentage of MVC in the swing phase of the gait cycle during inclined treadmill walking. RA, rectus abdominis; ES, erector spinae; RF, rectus femoris; BF, biceps femoris; VM, vastus medialis; TA, tibialis anterior; GCM, medial head of the gastrocnemius; SOL, soleus (*p < 0.05)

A correlation between net cardiopulmonary metabolic energy cost and lower-limb muscle activity was investigated during three inclined treadmill gait conditions (Fig. 4). The most significant positive correlation between net cardiopulmonary metabolic energy cost and muscle activity was seen in the SOL muscle in the stance phase during the 16% inclined treadmill gait (*r =* 0.487; *p* < 0.025). In addition, significant positive correlations were observed between net cardiopulmonary metabolic energy cost and muscle activation level in the GCM (*r =* 0.459; *p* < 0.0.036), RF (*r =* 0.443; *p* < 0.045), and VM (*r =* 0.436; *p* < 0.048) during the 16% inclined treadmill gait. The SOL, GCM, and VM muscle activity showed a positive correlation with the net cardiopulmonary metabolic energy cost at the relatively small inclination of 10% treadmill gait. In the swing phase, there were no muscles that exhibited a correlation with the net cardiopulmonary metabolic energy cost.

Muscles showing a significant relationship with an increase in the net cardiopulmonary metabolic energy cost during inclined walking were investigated using a linear regression model (net cardiopulmonary metabolic energy cost ~ %MVC + inclination + subject ID) (Table 3). In the stance phase, the SOL muscle activity change demonstrated the most significant relationship with an increase in the net cardiopulmonary metabolic energy cost. The VM, GCM, and RF muscle activity also displayed a significant relationship with an increased net cardiopulmonary metabolic energy cost.

**Table 3:** Relationship between the increase in net cardiopulmonary metabolic energy cost and changes in the percentage of MVC of the lower limb muscles during inclined treadmill walking

Muscle	Stance phase					Swing phase				
	*ß*	Std. Error	t	*p*	R^2^	*ß*	Std. Error	t	*p*	R^2^
RA	0.206	0.037	2.156	0.035^*^	0.377	0.084	0.047	0.820	0.415	0.377
ES	0.036	0.034	0.324	0.747	0.362	0.127	0.026	1.142	0.258	0.373
RF	0.164	0.026	1.648	0.104	0.395	0.121	0.029	1.202	0.234	0.384
VM	0.330	0.027	3.558	0.001^**^	0.469	0.108	0.035	1.085	0.282	0.381
BF	−0.088	0.030	−0.805	0.424	0.371	0.048	0.029	0.464	0.644	0.373
TA	0.017	0.055	0.159	0.874	0.378	0.018	0.030	0.181	0.857	0.378
GCM	0.318	0.028	3.216	0.002^*^	0.466	0.219	0.029	2.321	0.023^*^	0.417
SOL	0.375	0.030	3.774	0.000^***^	0.480	0.159	0.030	1.640	0.106	0.395

*RA* rectus abdominis, *ES* erector spinae, *RF* rectus femoris, *BF* biceps femoris, *VM* vastus medialis, *TA* tibialis anterior, *GCM* medial head of the gastrocnemius, *SOL* soleus

^*^p < 0.05, ^**^p < 0.01, ^***^p < 0.001

## Discussion

This study examined changes in the net cardiopulmonary metabolic energy cost and muscle activity patterns and assessed the relationship between changes in muscle activity and net cardiopulmonary metabolic energy cost among older adults for three inclined treadmill conditions. The results of this study revealed that net cardiopulmonary metabolic energy cost and most lower-limb muscle activity increased in this population as treadmill inclination increased. In particular, the RA, ES, RF, VM, BF, GCM, and SOL muscle activity was increased in the stance phase and the RA, ES, RF, VM, BF, and TA muscle activity was increased in the swing phase. Among the measured muscles, the ES activity showed the greatest increase in the stance and swing phases as the slope increased. There was a relationship between several types of muscle activity and the net cardiopulmonary metabolic energy cost. Specifically, the SOL muscle activity change showed the most significant correlation with an increase in the net cardiopulmonary metabolic energy cost.

Cardiopulmonary metabolic energy cost is an important variable in daily life and has been studied extensively. Also, the cardiopulmonary metabolic energy cost has become an important clinical tool for evaluating human exercise capacity and for predicting outcomes [18, 19]. The results of this study are consistent with those of previous studies showing that net cardiopulmonary metabolic energy cost expenditures increased significantly as the treadmill slope increased [7, 12]. In this study, walking under different slope conditions changed the net cardiopulmonary metabolic energy cost in older adults. Relative to the 0% inclined treadmill gait, the 10% inclined treadmill gait increased the net cardiopulmonary metabolic energy cost by 22.9%, while the 16% inclined treadmill gait increased the net cardiopulmonary metabolic energy cost by 44.2%.

In the previous studies of young adults, statistically significant changes were reported in the SOL and GCM muscle activities during inclined gait compared to flat gait [11, 12]. In contrast, elderly person demonstrated significant changes in the RF, VM, and BF in addition to the SOL and GCM muscles in our study. These results indicate that the elderly seems to use more lower-limb muscles than young adults during inclined gait. In addition, trunk and thigh muscles, such as RA and ES, were additionally measured in our study. Both muscles also showed significant changes during inclined gait compared to flat gait. Among the measured muscles, the ES activity showed the greatest increase in the stance and swing phases as the slope increased. Among the measured muscles, the ES activity showed the greatest increase in the stance and swing phases as the slope increased. Older subjects rely more strongly on excessive activity of their back muscles to regulate energy transfer during gait [9]. Previous studies have reported that back extensor muscle strength affects dynamic balance and postural control in older adults [20, 21]. Greater ES muscle activity in this study may imply that back muscle activity is increased during the whole gait cycle in older people so as to stabilize the trunk and to maintain a dynamic balance in accordance with increased treadmill inclination.

All measured muscles except TA showed significantly increased activity in the stance phase during inclined treadmill gait. In the swing phase, muscle activity was significantly increased in all measured muscles except GCM and SOL. Muscles whose activity did not significantly increase during a specific phase of the gait cycle did not have a primary function during the corresponding gait phase. From the mid-stance to terminal-stance stage, the calf muscle, SOL, and GCM showed significantly enhanced activity. Calf muscles have been frequently shown to be sources of strength in the trailing leg during the late-stance phase, used to propel the body’s center of mass anteriorly and to initiate swing in the anterior leg [22, 23]. In older adults, the plantar flexor power is weaker, causing weaker ground reaction force in this late-stance phase [24, 25]. In this study, walking under treadmill inclination conditions required older adults to display greater plantar flexor power as the treadmill inclination increased.

Muscular efficiency is one of the key determinants of the net cardiopulmonary metabolic energy cost of walking [26–28]. The results of this study showed high positive correlations between SOL and GCM activity and net cardiopulmonary metabolic cost. The correlations observed between net cardiopulmonary metabolic energy cost and ankle plantar flexor muscles in this study were similar to those reported in previous studies of young adults [11, 12, 29, 30]. In addition, the VM activity showed a positive correlation with the net cardiopulmonary metabolic energy cost. The VM forms a larger angle of insertion than the other muscles and provides medial stability to the patella [31]. Although the two knee extensors responded similarly under inclined treadmill gait conditions, we observed a more pronounced increase in VM than in RF activity with a steeper uphill grade. As compared with the biarticular RF, the uniarticular VM can be recruited more effectively as the knee extensor to selectively extend the knee during the initial contact to the mid-stance phase of inclined walking [32]. From initial contact to the mid-stance phase, an increase in VM activity seemed to lead to knee extension, which stabilized the knee joint as the slope increased.

Even though ES muscle activity was greatly increased in both the stance and swing phases during inclined treadmill gait, ES muscle activity was irrelevant in relation to the change in net cardiopulmonary metabolic energy cost. In contrast with the dynamic actions of the SOL, GCM, and VM muscles, the ES muscle showed isometric actions. These findings may support the notion that dynamic muscle actions consume more oxygen than isometric actions due to a large change in muscle length with contraction and relaxation [33]. In this study, the key muscles showing a high correlation with increased net cardiopulmonary metabolic energy cost during inclined treadmill gait were the SOL, GCM, and VM. To reduce the net cardiopulmonary metabolic energy cost and improve the efficiency of inclined walking, strengthening and ensuring efficient use of these muscles are very important in older adults.

One limitation of this study is a possible lack of robust generalizability due to our small sample size. Another limitation is that we did not include other gait parameters such as joint angle and moment in the analysis. Future studies are needed to confirm correlations between various gait parameters and net cardiopulmonary metabolic costs during inclined walking. Nevertheless, the results of this study can be used as basic data for designing walking-related training program and for the development of algorithms of wearable walking-assist robots for older adults.

## Conclusions

This study demonstrated that net cardiopulmonary metabolic cost and lower-limb muscle activity increase under inclined treadmill conditions in older adults. The key muscles showing a significant relationship with increased net cardiopulmonary metabolic energy cost during inclined treadmill gait were the SOL, GCM, and VM muscles. These muscles seemed to be important keys to controlling the net cardiopulmonary metabolic energy cost under inclined gait conditions in older adults.

## Data Availability

The datasets supporting the conclusions of this article are included within the manuscript.

## Abbreviations

sEMG: Surface electromyography
RA: Rectus abdominis
ES: Erector spinae
RF: Rectus femoris
BF: Biceps femoris
VM: Vastus medialis
TA: Tibialis anterior
GCM: Medial head of the gastrocnemius
SOL: Soleus
SPPB: Short physical performance battery
MVC: Maximum voluntary contraction
EEm: Energy expenditure measurement

## References

[1] Nations U. World population prospects 2019: highlights: Department of Economic and Social Affairs, Population Division; 2019.

[2] Mangione KK, Craik RL, McCormick AA, Blevins HL, White MB, Sullivan-Marx EM, Tomlinson JD (2010). Detectable changes in physical performance measures in elderly African Americans. Phys Ther.

[3] Manini T (2011). Development of physical disability in older adults. Curr Aging Sci.

[4] Mentiplay BF, Adair B, Bower KJ, Williams G, Tole G, Clark RA (2015). Associations between lower limb strength and gait velocity following stroke: a systematic review. Brain Inj.

[5] Giannotti E, Merlo A, Zerbinati P, Longhi M, Prati P, Masiero S, Mazzoli D (2015). Early rehabilitation treatment combined with equinovarus foot deformity surgical correction in stroke patients: safety and changes in gait parameters. Eur J Phys Rehabil Med.

[6] Studenski S, Perera S, Patel K, Rosano C, Faulkner K, Inzitari M, Brach J, Chandler J, Cawthon P, Connor EB (2011). Gait speed and survival in older adults. JAMA.

[7] Hortobágyi T, Finch A, Solnik S, Rider P, DeVita P (2011). Association between muscle activation and metabolic cost of walking in young and old adults. J Gerontol A.

[8] Toda H, Nagano A, Luo Z (2016). Age-related differences in muscle control of the lower extremity for support and propulsion during walking. J Phys Ther Sci.

[9] Lee H-J, Chang WH, Hwang SH, Choi B-O, Ryu G-H, Kim Y-H (2017). Age-related locomotion characteristics in association with balance function in young, middle-aged, and older adults. J Aging Phys Act.

[10] Padulo J, Powell D, Milia R, Ardigò LP (2013). A paradigm of uphill running. PLoS One.

[11] Kwee-Meier ST, Mertens A, Jeschke S (2018). Age-induced changes in the lower limb muscle activities during uphill walking at steep grades. Gait Posture.

[12] Silder A, Besier T, Delp SL (2012). Predicting the metabolic cost of incline walking from muscle activity and walking mechanics. J Biomech.

[13] Volpato S, Cavalieri M, Sioulis F, Guerra G, Maraldi C, Zuliani G, Fellin R, Guralnik JM (2011). Predictive value of the short physical performance battery following hospitalization in older patients. J Gerontol A.

[14] Brockway J (1987). Derivation of formulae used to calculate energy expenditure in man. Hum Nutr Clin Nutr.

[15] Merletti R, Hermens H (2000). Introduction to the special issue on the SENIAM European concerted action. J Electromyogr Kinesiol.

[16] Perry J, Davids JR (1992). Gait analysis: normal and pathological function. J Pediatr Orthop.

[17] Giroux M, Moissenet F, Dumas R (2013). EMG-based validation of musculo-skeletal models for gait analysis. Comput Methods Biomech Biomed Eng.

[18] Gupta SD, Bobbert MF, Kistemaker DA (2019). The metabolic cost of walking in healthy young and older adults–a systematic review and Meta analysis. Sci Rep.

[19] Tung KD, Franz JR, Kram R (2014). A test of the metabolic cost of cushioning hypothesis during unshod and shod running. Med Sci Sports Exerc.

[20] Granacher U, Lacroix A, Muehlbauer T, Roettger K, Gollhofer A (2013). Effects of core instability strength training on trunk muscle strength, spinal mobility, dynamic balance and functional mobility in older adults. Gerontology.

[21] Shahtahmassebi B, Hebert JJ, Hecimovich M, Fairchild TJ (2019). Trunk exercise training improves muscle size, strength, and function in older adults: a randomized controlled trial. Scand J Med Sci Sports.

[22] Neptune RR, Clark DJ, Kautz SA (2009). Modular control of human walking: a simulation study. J Biomech.

[23] Bohm S, Mersmann F, Santuz A, Arampatzis A (2019). The force–length–velocity potential of the human soleus muscle is related to the energetic cost of running. Proc R Soc B.

[24] Boyer KA, Johnson RT, Banks JJ, Jewell C, Hafer JF (2017). Systematic review and meta-analysis of gait mechanics in young and older adults. Exp Gerontol.

[25] Franz JR, Kram R (2013). Advanced age affects the individual leg mechanics of level, uphill, and downhill walking. J Biomech.

[26] Cavagna G, Kaneko M (1977). Mechanical work and efficiency in level walking and running. J Physiol.

[27] Donelan JM, Kram R, Kuo AD (2002). Mechanical work for step-to-step transitions is a major determinant of the metabolic cost of human walking. J Exp Biol.

[28] Vernillo G, Giandolini M, Edwards WB, Morin J-B, Samozino P, Horvais N, Millet GY (2017). Biomechanics and physiology of uphill and downhill running. Sports Med.

[29] Ortega JD, Farley CT (2015). Effects of aging on mechanical efficiency and muscle activation during level and uphill walking. J Electromyogr Kinesiol.

[30] Saito A, Tomita A, Ando R, Watanabe K, Akima H (2018). Similarity of muscle synergies extracted from the lower limb including the deep muscles between level and uphill treadmill walking. Gait Posture.

[31] Bejarano NC, Pedrocchi A, Nardone A, Schieppati M, Baccinelli W, Monticone M, Ferrigno G, Ferrante S (2017). Tuning of muscle synergies during walking along rectilinear and curvilinear trajectories in humans. Ann Biomed Eng.

[32] Franz JR, Kram R (2012). The effects of grade and speed on leg muscle activations during walking. Gait Posture.

[33] Elder CP, Mahoney ET, Black CD, Slade JM, Dudley GA (2006). Oxygen cost of dynamic or isometric exercise relative to recruited muscle mass. Dyn Med.

